# Over-the-scope clip-assisted endoscopic full-thickness resection has potential to treat complex nonampullary duodenal lesions: a single-center case series

**DOI:** 10.1186/s12876-021-02068-x

**Published:** 2021-12-16

**Authors:** Yongqiu Wei, Qiaozhi Zhou, Ming Ji, Shutian Zhang, Peng Li

**Affiliations:** Department of Gastroenterology, Beijing Friendship Hospital, National Clinical Research Center for Digestive Diseases, Beijing Digestive Disease Center, Beijing Key Laboratory for Precancerous Lesion of Digestive Diseases, Capital Medical University, No. 95, Yongan Road, Xicheng District, Beijing, 10050 China

**Keywords:** OTSC, OTSC-assisted endoscopic full-thickness resection, Endoscopic full-thickness resection, Nonampullary duodenal lesions

## Abstract

**Background and aims:**

The duodenum is considered a challenging area for the endoscopic resection of lesions. This study aimed to evaluate the efficacy and safety of over-the-scope clip (OTSC)-assisted endoscopic full-thickness resection (EFTR) for complex nonampullary duodenal lesions unsuitable for conventional resection techniques.

**Methods and patients:**

We conducted a retrospective case review of 13 consecutive patients with complex nonampullary duodenal tumors that were unsuitable for conventional resection techniques; these patients underwent EFTR assisted with OTSC at Beijing Friendship Hospital, Capital Medical University from September 2015 to September 2020. The OTSC device was placed, and tumors were resected after the lesions were identified. Data were abstracted for demographics, lesion features, histopathologic diagnoses, technical success rates, complete resection (R0 resection) rates, and complications.

**Results:**

Thirteen patients with duodenal lesions (6 adenomas and 7 submucosal tumors with nonlifting signs, incomplete lifting signs, difficult locations, failed ESD/EMR attempts or suspected origin in the muscularis propria) subjected to EFTR were included. The sizes of all the lesions evaluated by endoscopy were smaller than 20 mm, and most of them (84.6%, 11/13) were smaller than 12 mm. All 13 applications of the clips, endoscopic resection and full-thickness resection were successful (13/13, 100%). Complete resection was achieved in 12 patients (12/13, 92.3%). There were no immediate or delayed complications, including bleeding, infection and perforation.

**Conclusions:**

OTSC -assisted EFTR appears to be effective and safe for complex nonampullary duodenal lesions smaller than 20 mm (particularly those ≤ 10–12 mm) that are unsuitable for conventional resection techniques.

## Introduction

For duodenal tumors, traditional endoscopic treatment, such as endoscopic mucosal resection (EMR) and endoscopic submucosal resection (ESD), has not been carried out as smoothly as in the stomach, esophagus and colon because the anatomical structure of the duodenum has brought endoscopic treatment more difficulties and challenges, such as the narrow lumen and Brunner’s glands in the submucosa, which harden the duodenal wall and result in poor mucosal lifting. The thin muscle layer leads to a higher incidence of perforation and bleeding [1, 2]. Furthermore, when emergency surgery is required, the precipitous flexure of the duodenum may not provide the necessary stability, and it is difficult to accurately reach the target [1, 3].

In recent years, research on endoscopic full-thickness resection (EFTR) allowing for definitive diagnosis and potential curative treatment of lesions involving any layer of the gastrointestinal wall has gradually increased, which may help solve this problem. There are three kinds of methods for EFTR. Conventionally exposed EFTR first adopts EFTR accompanied by artificial perforation and then uses the Overstitch System (Apollo Endosurgery, Austin, USA) or the over-the-scope clip (OTSC) device (Ovesco Endoscopy, Tübingen, Germany) to close the wound, thereby posing the risk of exposure to abdominal cavity infection [4–6]. The new full-thickness resection device (FTRD; Ovesco Endoscopy, Tübingen, Germany) system is designed in such a way that it allows complete and reliable closure of the gastrointestinal wall by applying an OTSC immediately before snare resection of the enclosed gut wall, which achieves a nonexposed EFTR, limited by oversize and poor flexibility in the upper gastrointestinal tract. Consequently, the FTRD device is currently approved only for use in the lower gastrointestinal tract. The third type of EFTR is similar to FTRD, in which an OTSC is applied first, followed by full-thickness resection after the location of the lesion is confirmed. The third EFTR method (assisted with OTSC) has good flexibility and convenient operation and seems more suitable for duodenal lesions. In comparison with simple OTSCs or overstitch closure after full-thickness resection, no free perforation of the gut occurs at any time during the latter two EFTR procedures [4, 6–8].

A few case reports and case series have been published on the first two EFTR methods [5, 9], but successful OTSC-assisted EFTR in duodenal lesions has been published in few reports to date—approximately 31 cases in total (Table 1) [10–18], and most of these studies were exploratory attempts to treat non-difficult lesions; thus, conventional ESD or EMR may still have a high success rate. Here, we report 13 case series of OTSC-assisted EFTR for complex nonampullary duodenal lesions (with nonlifting signs, incomplete lifting signs, difficult location, suspected origin in the muscularis propria or failed ESD/EMR attempts) at our medical center. To the best of our knowledge, this is the first and largest case series of OTSC-assisted EFTR for complex nonampullary duodenal lesions to date.

**Table 1:** Summary of studies in the present review

References	Indication	No. of patients	Lesion size (mm), median (range)	Technical success rate (%)	Complications	R0 rate (%)
Fahndrich et al. [10]	NET	1	20	100(1/1)	No	100 (1/1)
Schmidt et al. [11]	SMT (2), Nonlifting adenoma (2)	4	SMT, 16 (10-22) Adenoma, 22.5 (15-30)	100 (4/4)	Minor bleeding in 2 patients	75 (3/4)
Milano et al. [16]	NET	1	10	100 (1/1)	No	100 (1/1)
Sarker et al. [15]	SMT	3	11.3 (9-15)	100 (3/3)	No	100 (3/3)
Al-Bawardy et al. [12]	SMT	3	9.3 (5-15)	100 (3/3)	No	100 (3/3)
Schempf et al. [14]	R1-margins of NET (G1)	1	15	100 (1/1)	No	100 (1/1)
Andrisani et al. [13]	Nonlifting sign, SMT, Adenoma recurrence	4	14.3 (10-20)	100 (4/4)	No	75 (3/4)
Nassri et al. [17]	NET	1	10	100 (1/1)	No	100 (1/1)
Tashima et al. [18]	NET	13	6 (3-8)	100 (14/14)	No	92.9 (13/14)

SMT, submucosal tumor; NET, neuroendocrine tumor

## Methods and patients

### Patient information

Between September 2015 and September 2020, 13 patients underwent complex nonampullary duodenal lesion EFTR assisted with OTSC at the Department of Gastroenterology in Beijing Friendship Hospital, Capital Medical University. The indications of EFTR included the following conditions: (1) EUS suspects that the lesion originated in the muscularis propria or submucosa; (2) The location of the lesion makes it difficult to perform conventional EMR or ESD operations, such as the bulb-descending junction, posterior wall of the bulb, or other parts where maintaining a stable endoscope and clear vision is challenging; (3) The lifting sign is negative or incomplete, the attempt to perform ESD or EMR treatment fails, or the patient refuses surgery and cannot accept the increased risks of EMR and ESD and is willing to try the EFTR strategy after detailed communication of the condition. When more than two of the above conditions occur, EFTR treatment will be considered. The exclusion criteria were as follows: (1) The preoperative assessment of the lesion is too large (usually the lesion needs to be ≤ 20 mm), and the endoscopist considers it challenging to clamp the base of the lesion with OTSC; (2) The patient refused endoscopic treatment.

Endoscopic ultrasound (EUS) was performed in all 13 patients before resection, confirming the origin and nature of the lesions and detecting that no important surrounding tissue structures are involved, such as the pancreaticobiliary system, blood vessels, and intestines. Before the operation, it is routinely recommended to perform enhanced computed tomography (CT) of the abdomen and submucosal injection to assist in assessing the origin of the lesion. Written informed consent was obtained from all 13 patients. Data were collected retrospectively. The main outcome measures were technical success, R0 resection, histologic confirmation of full-thickness resection, and complication events. The study was conducted in accordance with the Declaration of Helsinki and was approved by the Bioethics Committee of Beijing Friendship Hospital, Capital Medical University (IRB no. 2018-P2-191-01).

### EFTR procedure and postoperative management

**Fig. 1:**
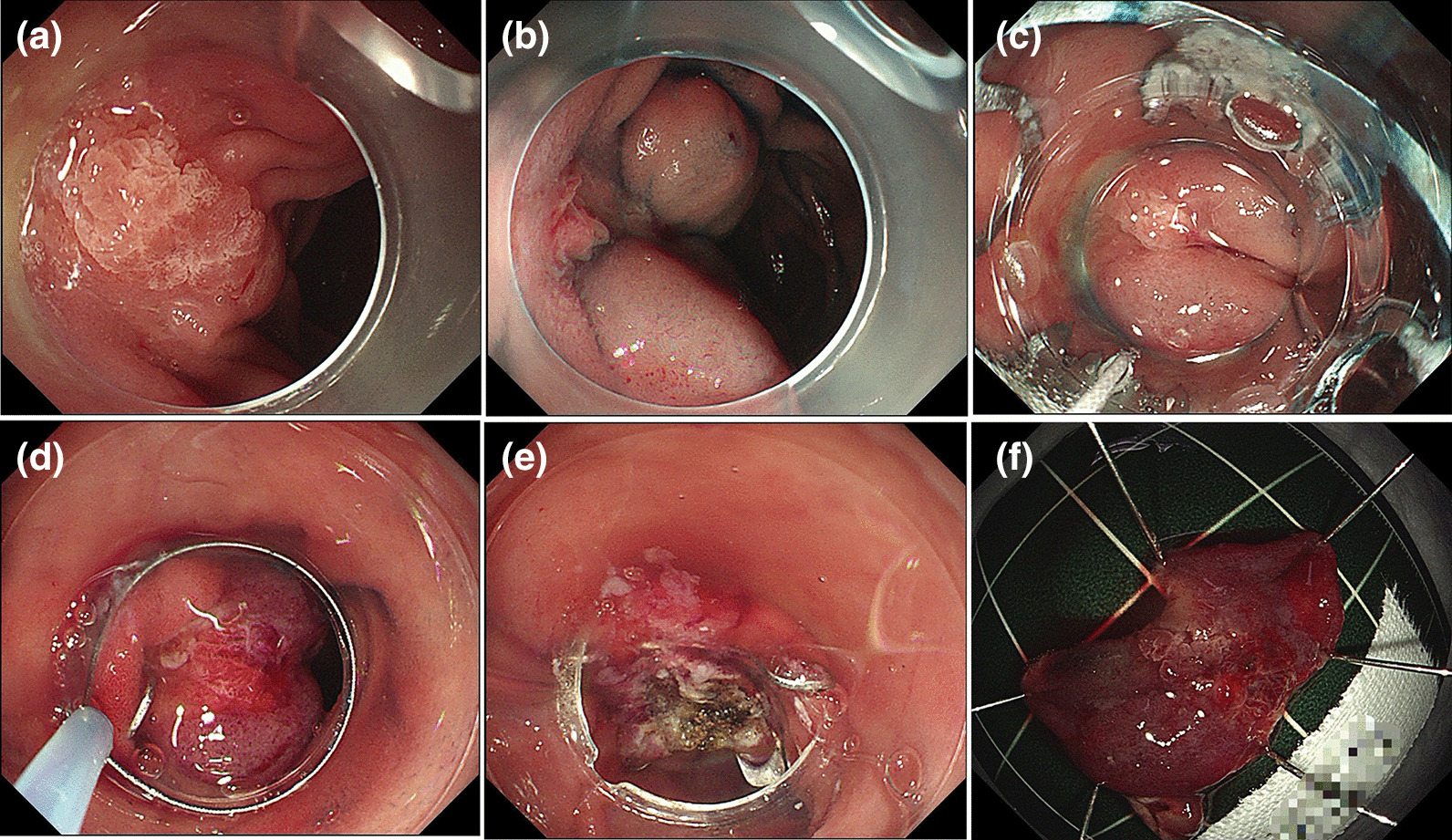
Endoscopic full-thickness resection (EFTR) procedure for an adenoma in the descending part of the duodenum. **a** Identification of the duodenal lesion. **b** Incomplete lifting sign. **c** The clip was placed on the endoscope and introduced to catch the lesion. **d** The clip was deployed, creating a pseudopolyp of the lesion above the closed clip. Hereafter, the target lesion was resected en bloc above the clip using an electrosurgical snare. **e** Resection defect. **f** En bloc resected specimen

**Fig. 2:**
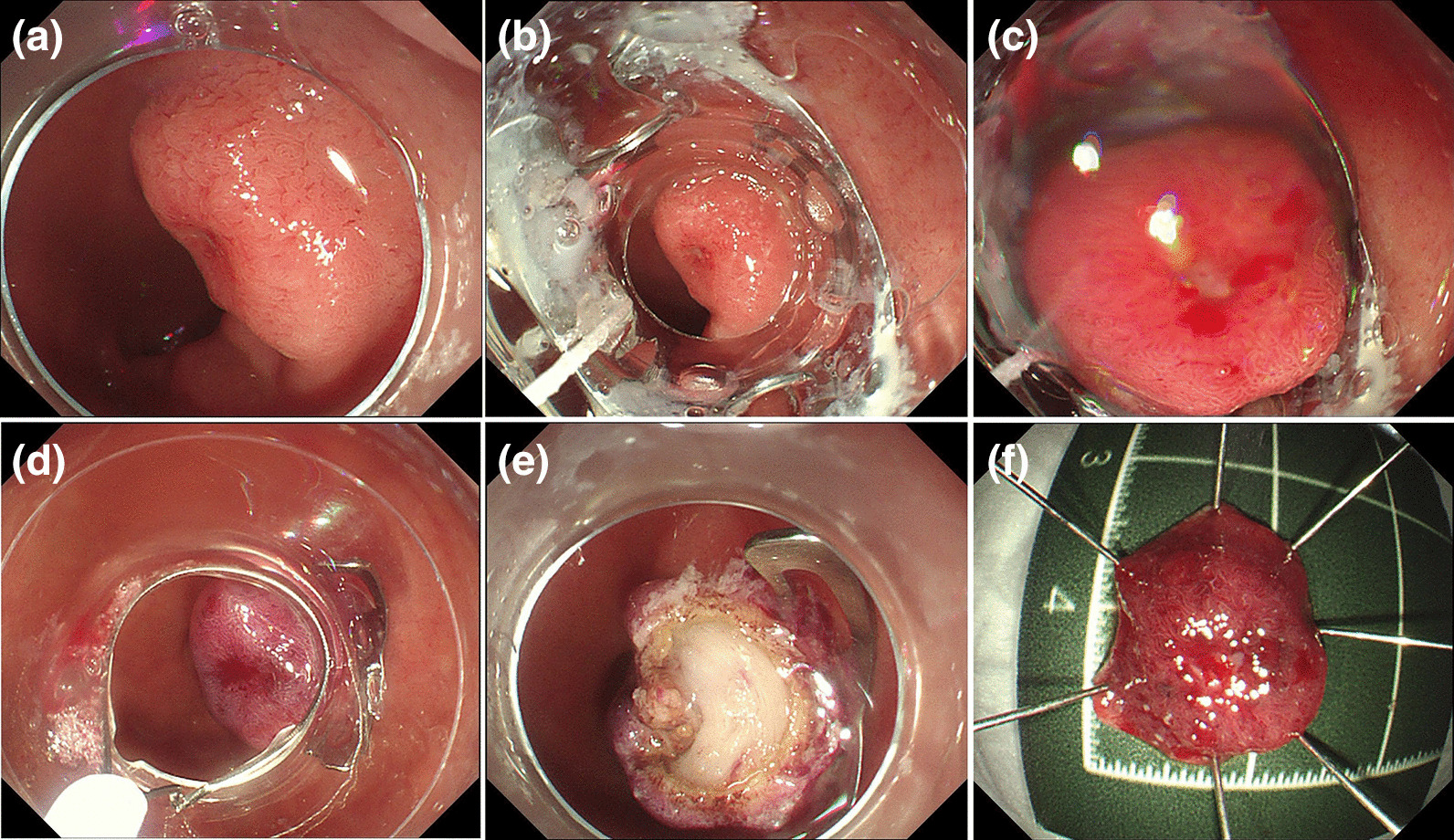
Endoscopic full-thickness resection (EFTR) procedure for a submucosal tumor in the posterior wall of the bulb-descending junction. **a** The submucosal tumor had a superficial ulcer located in the posterior wall of the bulb-descending junction. **b** The clip was placed on the endoscope and introduced to catch the lesion. **c** The clip was deployed, creating a pseudopolyp of the lesion above the closed clip. **d** The target lesion was resected en bloc above the clip using an electrosurgical snare. **e** Resection defect. **f** En bloc resected specimen

The OTSC-assisted EFTR procedure was a multistep process performed roughly as previously described [12]. After the lesion was confirmed, the OTSC device was preassembled on a transparent cap over the gastroscope (GIF-Q260J; Olympus), which is used routinely. When the lesion is difficult to inhale into the transparent cap, a double-channel gastroscope (GIF-2T240; Olympus) combined grasper will be selected. When the lesion is located on the anal side of the duodenal papilla, CF-HQ290I (Olympus) may be selected. The lesion was fully aspirated into the transparent cap, and the clip was deployed. Thus, the target lesion was situated on the created pseudopolyp above the closed clip. We used an 18.1-mm diameter transparent cap (D-206; Olympus) with a 25-mm electrosurgical snare (SD-221 L-25; Olympus) preset to suck the target lesion into the cap and gradually release the snare to capture the lesion. When the lesion is completely captured, the snare is gradually shrunken and the lesion is gently lifted to reduce the current spreading along the clip causing unnecessary damage. The pseudopolyp over the clip was subsequently resected en bloc with the electrosurgical snare. Thereupon, the OTSC was placed, and the lesions were resected after they were identified (Figs. 1, 2). We prepared clips with working channel diameters of 11, 12, and 14 mm according to the lesion size because the maximum outer diameters of these application caps are 16.5 mm, 17.5 mm, and 21 mm, respectively, indicating that lesions smaller than this range may be sealed. Finally, all 13 patients completed EFTR using clamps with working channel diameters of 11 and 12 mm.

All procedures were performed by two dedicated therapeutic endoscopists (L. P and J. M), who had previous experience with the OTSC device system and more than 60 cases of OTSCs per endoscopist. The postoperative management were performed roughly as previously described [5, 15]. All patients were maintained under general anesthesia during the procedure. After the EFTR, patients had to fast for 48-72 h. Proton pump inhibitors were routinely administered intravenously. These patients were under observation for abdominal pain, fever, and signs of peritonitis or hemorrhage. If no complications occurred, these patients were discharged from the hospital 48-72 h postoperatively; standard follow-up by esophagogastroduodenoscopy was recommended.

### Endpoints

We aimed to assess the technical feasibility, R0 resection rate, full-thickness resection rate, and complication events. Technical success was defined as appropriate deployment of the OTSC and successful resection of the lesion. Full-thickness resection was defined as the presence of muscularis propria in the resected specimen. R0 was defined as microscopic free lesion margins in the specimen. Typically, the tumor-free range on the edge of the lesion is ≥ 1 mm.

## Results

Thirteen patients (7 men, 6 women; median age: 51±9.3 years; range 42–75 years) with nonampullary duodenal lesions underwent the OTSC-assisted EFTR procedure. The indications included 6 mucosal lesions and 7 submucosal lesions with nonlifting signs, incomplete lifting signs, and difficult locations, failed ESD/EMR attempts or suspected origin in the muscularis propria. The mean size of the endoscopic estimate was 9.4 ± 3.2 mm (range 5–15) for submucosal lesions and 11 ± 4.4 mm (range 6–20) for mucosal lesions.

The mean procedure time was 38.7 ± 14.6 min (range 12–67). The mean size of the resected specimen by histology was 16 ± 5.3 mm (range 10–26) for submucosal lesions and 24 ± 6 mm (range 15–32) for mucosal lesions. Postresection histopathologic diagnoses of the duodenal lesions included neuroendocrine tumors (n = 6), gastrointestinal stromal tumors (n = 1), adenomas with high-grade dysplasia (HGD) (n = 4) and adenomas with low-grade dysplasia (LGD) (n = 2).

All 13 applications of the clips and endoscopic resection were successful (13/13, 100%). Full-thickness resection and en bloc resection were achieved in all patients (13/13, 100%), complete resection (R0 resection) was achieved in 12 patients (12/13, 92.3%), and the other patient had a suspicious tubular adenoma at the pathological margin. No immediate or delayed complications, including but not limited to bleeding, infection or perforation, were noted during a median follow-up of 811 days (range 90–1804 days). The characteristics of the patients and the results of OTSC-assisted EFTR are shown in Table 2.

**Table 2:** Patient characteristics and outcomes following over-the-scope clip-assisted endoscopic full-thickness resection

#	Age/sex	Location	Indication	Lesion size (endoscopic estimate) (mm)	Resected specimen size (mm)	Technique success	EFTR	Complications	R0	Preoperative biopsy	Final pathology	Operation time (min)
1	43/M	Anterior wall of the bulb	Difficult location, submucosal lesion and nonlifting sign	5	10	Yes	Yes	None	Yes	NET(G1)	NET(G1)	57
2	62/M	Anterior wall of the bulb	Suspected in MP and nonlifting sign	10	15	Yes	Yes	None	Yes	None	NET(G1)	30
3	42/F	Greater curve of the bulb	Suspected in MP, difficult location, incomplete lifting sign and failed ESD attempt	10	17	Yes	Yes	None	Yes	None	NET(G2)	53
4	53/M	Descending part	Adenoma with HGD, difficult location and nonlifting sign	12	31	Yes	Yes	None	Yes	Adenoma with HGD	Adenoma with HGD	38
5	54/M	Descending part	Adenoma, incomplete lifting sign, difficult location and failed EMR attempt	20	25	Yes	Yes	None	Yes	Adenoma with LGD	Adenoma with LGD	67
6	62/F	Descending part	Difficult location, suspected in MP and nonlifting sign	15	20	Yes	Yes	None	Yes	None	Gist	25
7	57/F	Descending part	Adenoma, difficult location and incomplete lifting sign	12	22	Yes	Yes	None	Yes	Adenoma with LGD	Adenoma with LGD	40
8	46/F	Descending part	Difficult location, submucosal lesion	12	26	Yes	Yes	None	Yes	NET(G1)	NET(G1)	27
9	57/M	Descending part	Adenoma with HGD, difficult location and incomplete lifting sign	8	32	Yes	Yes	None	No	Adenoma with HGD	Adenoma with HGD	27
10	51/M	Descending part	Adenoma with HGD and incomplete lifting sign	10	17	Yes	Yes	None	Yes	Adenoma with HGD	Adenoma with HGD	47
11	46/F	Posterior wall of the bulb-descending junction	Difficult location and suspected in MP	8	14	Yes	Yes	None	Yes	None	NET(G1)	12
12	51/F	Anterior wall of the bulb	Difficult location, submucosal lesion and incomplete lifting sign	6	10	Yes	Yes	None	Yes	None	NET(G2)	33
13	75/M	Greater curve of the bulb-descending junction	Adenoma with HGD, difficult location and incomplete lifting sign	6	15	Yes	Yes	None	Yes	Adenoma with HGD	Adenoma with HGD	47

M male, F female, NET, neuroendocrine tumor; HGD high-grade dysplasia; LGD low-grade dysplasia; Gist gastrointestinal stromal tumors; MP, muscularis propria

Eight patients underwent follow-up endoscopy, including the patient with suspected tubular adenoma at the pathological margin. The median time to follow-up endoscopy was 357 days (range 31–1277 days). All 8 endoscopic follow-up examinations with biopsy sampling were negative for residual disease or recurrence. Among the 8 patients who underwent follow-up endoscopy, the clips were retained in place in four patients.

The other 5 patients who did not undergo endoscopic follow-up in our hospital returned to their local hospital (which had recommended them to our facility for EFTR) and were there followed up under endoscopy. No local residual lesions or recurrence was found, and no more detailed information was available.

## Discussion

Duodenal tumors, including mucosal tumors and submucosal tumors, are a relatively rare and mostly coincidental finding in patients undergoing upper gastrointestinal endoscopy compared to other areas of the gastrointestinal tract. With the development and popularization of endoscopic techniques, the incidence of duodenal tumors has gradually increased. We realized that this disease, which may cause significant mortality, has actually been in existence before, but in many cases, we could not find it. Located in the retroperitoneum, the duodenum is very fragile, with a relatively thinner wall than that in the rest of the digestive tract, and Brunner’s glands in the submucosa harden the duodenal wall, occasionally resulting in poor mucosal lifting. Furthermore, the existence of abundant blood vessels and narrow lumens increases the incidence of complications [3, 5, 19].

Duodenal endoscopic resection techniques such as EMR and ESD are feasible and effective as therapeutic procedures, but they also have important limitations. The risk of perioperative complications such as delayed bleeding, infection, and perforation has been repeatedly reported [20, 21]. EMR is technically less difficult for epithelial tumors smaller than 10–15 mm, but it is also disadvantageous in terms of the R0 resection rate and complication rate. In contrast, the R0 resection rate is higher for ESD, but the risk of perforation is more than 30% with ESD in the duodenum [20, 22]. In particular, under certain conditions, such as nonlifting adenomas, submucosal lesions, or lesions originating in the muscularis propria, ESD is challenging and harbors a significant risk of adverse events without using conventional techniques [1, 2, 23]. When it is difficult to maintain a stable position of the endoscope and a clear field of view (such as that of the bulb-descending junction, the posterior wall of the bulb, or other parts where it is difficult to maintain a stable endoscope), the traditional endoscopic treatment strategy is more challenging. Nevertheless, surgical excision treatment, such as Whipple’s pancreatectomy, pancreas-preserving duodenectomy, and pylorus-preserving pancreatectomy, is too aggressive and invasive and is accompanied by serious surgical risks and significant mortality [2, 5, 24]. The choice of treatment options is even more troublesome, especially when the lesion is only precancerous or early cancer without a risk of lymph node metastasis. Recently, EFTR, which can provide a complete basis for pathological evaluation, has emerged as an option to solve some difficult lesions that are not optimally treated by standard resection methods. Hence, OTSC device-assisted EFTR might become a minimally invasive alternative strategy for these selected patients.

The OTSC is a novel device that was developed for the treatment of gastrointestinal bleeding, perforations, leaks or fistulas. After resection of some larger or deeper lesions, the OTSC device was deployed to close a potential or existing perforation [25]. OTSC-assisted EFTR is a “close-then-cut” nonexposed EFTR that is safer than “cut-then-close” EFTR in that the former avoids contamination of gastrointestinal luminal content into the peritoneum. There have been only a few case reports about OTSC-assisted duodenal tumor EFTR followed by resection of a pseudopolyp using snare resection or a needle knife (Table 1) [4, 10–18]. Most of the case reports published in the past were technical exploratory treatments for non-difficult lesions; thus, conventional ESD or EMR may still have a high success rate. However, these previous case reports also confirmed the feasibility of the technical solution applied here, and provide a supportive basis for our current research. In our study, the freedom to combine larger transparent caps, snares, and a double-channel gastroscope-combined grasper as necessary creates better a condition for the successful application of the process.

A dedicated FTRD (Ovesco Endoscopy) that consists of an OTSC preloaded into a cap with an integrated snare has been approved for colonic use in the United States, and a few case series investigations and applications in the stomach and duodenum have been applied for lesions not amenable to EMR or ESD [26–29]. Compared with the aforementioned OTSC-assisted EFTR, the FTRD is limited in regards to visualization through the scope, and its larger size (with an outer diameter of 21 mm) makes it difficult to advance through the upper esophageal sphincter or pyloric ring into the duodenum and usually requires through-the-scope balloon dilation to aid passage of the device, increasing the risk of tearing, perforation and bleeding [8, 13, 29].

In our series, 13 patients who were unsuitable for traditional endoscopic treatment strategies because of nonlifting signs, incomplete lifting signs, difficult location, suspected origins in the muscularis propria, or failed ESD/EMR attempts finally received EFTR treatment. We used commercially available OTSC devices and an electrosurgical snare to assist the EFTR procedure. Considering that the size of the resected lesions is often limited by the size of the OTSC cap selected, the maximum diameter of all the lesions in our case series were less than 20 mm, most of them were less than 12 mm (11/13, 85%), and all OTSC caps were used with a working diameter of 11 or 12 mm (the maximum outer diameters of these application caps are 16.5 mm and 17.5 mm). We already know from clinical practice and literature reports that the FTRD is often difficult to deliver to the stomach, despite the use of balloon expansion. In the limited number of case reports, the technical success rate of the FTRD in the duodenum is generally approximately 66.7–85.0% [8, 13, 29]. In contrast, in our case series study, both the technical success rate and the full-thickness resection rate reached 100% (13/13). Not only did no surgical complications occur, but the operation time was also shorter than that in the existing, limited EFTR studies [8, 29]. Indeed, OTSC-assisted EFTR using a commercially available OTSC device seems to be more convenient and flexible than the FTRD for complex lesions in the narrow duodenum. In addition, although the lesions were of more complex types, that is, lesions with poorly lifting signs, difficult locations, suspected origins in the muscularis propria, or failed ESD/EMR attempts, the R0 resection rate reached 92.3% (12/13), which is higher than that in the related reports of FTRD-EFTR (63.2-83.0%) and is also difficult for EMR and ESD to beat [1, 8, 20–23]. However, in our study, all lesions evaluated by endoscopy were smaller than 20 mm, and approximately 84.6% (11/13) and 61.5% (8/13) were smaller than 12 mm and 10 mm, respectively. To ensure complete resection, the target lesion should be below 20 mm (under 10–12 mm is better) for OTSC-assisted EFTR in the duodenum. We speculate that the procedure may be effective for lesions between 12 and 20 mm in size, based on the results obtained in 31 published cases (Table 1). The conclusion remains to be further confirmed in a future study with a large sample. Although this technique will not replace existing methods, it may become a useful addition to the therapeutic armamentarium of interventional endoscopists. In addition, the price of this device is very expensive, probably approximately 20,000 RMB per device, which will restrict its wide application.

OTSCs are made from nitinol, which has good biocompatibility and was originally considered a permanent implant material for endoscopic treatment [30]. Although the safety and effectiveness of OTSCs in endoscopic treatment have been confirmed in the past 10 years, the safety of long-term OTSC retention in the human body has not been confirmed on a large scale. Unlike ordinary titanium clips, OTSCs do not readily detach from the mucosa spontaneously. At present, the retention of the OTSC in the human body is under clinical observation and follow-up. In rare cases, such as when repeated biopsy or therapy is required, the OTSC is misplaced, poor healing occurs, adverse events arise after OTSC implantation and on patient request, OTSC removal can be attempted [30]. During our follow-up of 4 patients, spontaneous OTSC detachment was not observed. The patients’ tumors were completely removed, there were no signs of recurrence during follow-up, and there was no urgent need for OTSC removal. However, in the long term, implanted OTSCs may spontaneously detach.

There are several limitations in our present investigation. First, this was a retrospective, single-center study, and the results are not generalizable to all patients who have undergone complex nonampullary duodenal EFTR. Second, we had a small sample size; thus, larger prospective studies are required to reinforce our primary results. In addition, all procedures were performed by two experienced and skilled endoscopists in our center, and consequently, the findings of subsequent studies in other centers may vary depending on the experience and skills of different endoscopists.

In summary, OTSC-assisted EFTR to treat challenging nonampullary duodenal lesions smaller than 20 mm (particularly for lesions ≤ 10–12 mm) appears to be safe, effective and fairly easy. This method expands the currently available endoscopic resection techniques. More prospective studies evaluating the efficacy and safety of nonampullary duodenal resections comparing this technique with existing methods are warranted.

## Data Availability

The datasets generated and analyzed during the current study are available from the corresponding author upon reasonable request.

## Abbreviations

OTSC: Over-the-scope clip
EFTR: Endoscopic full-thickness resection
EMR: Endoscopic mucosal resection
ESD: Endoscopic submucosal resection
FTRD: Full-thickness resection device
EUS: Endoscopic ultrasound
HGD: High-grade dysplasia
LDG: Low-grade dysplasia
SMT: Submucosal tumor
NET: Neuroendocrine tumor
Gist: Gastrointestinal stromal tumors
MP: Muscularis propria
